# Use of Citizen Science to Determine Prey Partitioning Between Two Coastal Raptors

**DOI:** 10.1002/ece3.72518

**Published:** 2025-12-26

**Authors:** Leo Biggs, Greg S. Baxter, Stephen J. S. Debus, Neal Finch, Anysha Riggs, Hayden Houweling, Melissa Appleby, Peter J. Murray

**Affiliations:** ^1^ School of Agriculture and Environmental Science University of Southern Queensland Queensland Australia; ^2^ Zoology, School of Environmental and Rural Science University of new England Armidale New South Wales Australia

**Keywords:** citizen science, interspecies competition, niche overlap, prey partitioning, raptor diet, social media

## Abstract

In order to effectively conserve a species, it is often important to understand the ecological niche it occupies, and the resources required to sustain it. For predators, prey is typically the most important resource, and predators either outcompete for or partition resources to secure long‐term food security. Raptors have been shown to utilise both strategies. However, demonstrating competition and prey partitioning between raptors is difficult. Traditional diet collection methods can be prohibitively time‐consuming over large spatial areas and biased towards breeding individuals and nest sites. Use of citizen science, in particular images and videos recorded from social media, potentially overcomes these issues. This theory was tested by investigating the dietary breadth, diversity, overlap and competition between two Australian raptors, the Eastern Osprey (*Pandion haliaetus cristatus*) and White‐bellied Sea‐Eagle ( *Haliaeetus leucogaster* ) (WBSE), in New South Wales and Queensland (Australia). These raptors were suspected of occupying the same realised niche and partitioning the fish in their diets to reduce direct competition and conflict. The fish component of both raptor diets was shown to be moderately broad and diverse, with greater evenness in the diet of WBSE, and an Eastern Osprey preference for mullet (Mugilidae). There was a moderate dietary overlap, with Morisita's Index values of between 0.59 and 0.61. Competition between the raptors was assessed to be low due to the high abundance of shared prey, and limited overlap in other prey. Prey partitioning appeared to be determined by resource availability. Highly abundant prey is shared, whilst less abundant prey is partitioned according to hunting style, with WBSE swooping to collect fish from the water surface, and Eastern Osprey diving to access deeper dwelling fish. This study represents the first attempt to study a complex dietary relationship in raptor prey partitioning using diets sourced from social media. The data provided adequate detail to demonstrate dietary partitioning and identify potential causes.

## Graphical abstract

In Australia, Eastern Osprey ( *Pandion haliaetus* cristatus) and White‐bellied Sea‐Eagle ( *Haliaeetus leucogaster* ) occupy the same ecological niche and are suspected of partitioning prey resources. This study investigated this through evaluating the breadth, overlap and partitioning of diets recorded from social media posts. Eastern Osprey and White‐bellied Sea Eagles were found to share highly abundant prey, while less abundant prey is partitioned according to hunting style.

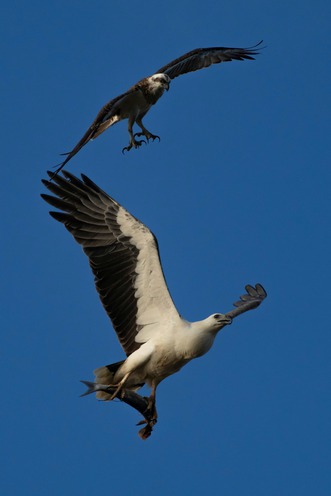

## 1 Introduction

The ecological niche of a species describes how that species interacts with its environment, detailing the habitat it utilises, the resources it consumes, and how it interacts with its own or other species within the same community (Chase and Leibold 2003). It is important to understand both current and historical ecological niches when looking to make threatened species assessments and conservation recommendations (Britnell et al. 2023). For predators, one of the primary resources of interest is prey. Predators compete with each other by interference (i.e., aggressive exclusion) (Case and Gilpin 1974), intraguild predation (i.e., preying on competing predators) (Holt and Polis 1997) or by evolving specialised hunting strategies (Michalko and Pekár 2016). Resource partitioning can prevent the demise of outcompeted predators, and resource partitioning studies determine the limits placed on species coexistence by competition (Schoener 1974). For predators, resource partitioning is achieved by altering the time of day in which they hunt, specialising to occupy different areas within the niche or by utilising different food types (Schoener 1974). The partitioning of prey can be measured through analysing niche overlap. Niche overlap refers to the overlap in resources utilised by two or more species that occupy the same environment (Hurlbert 1978). The acceptable level of niche overlap to sustain multiple predators is dependent on the abundance of prey, with species competing less for highly abundant resources, allowing a greater acceptable level of overlap (Pianka 1974).

Raptors, members of the Accipitriformes, Falconiformes, Strigiformes, Cathartiformes and Cariamiformes (McClure et al. 2019), are a group of avian predators that include over 550 species, 52% of which have declining populations and 18% are listed as threatened by BirdLife International (McClure et al. 2018). Understanding of ecological niches and interspecies competition has been sought to improve the conservation outcomes of threatened species of condors and vultures (Anoop et al. 2020; Ballejo et al. 2018; D'Elia et al. 2015), eagles (Carrete et al. 2002; Sutton et al. 2024; Väli et al. 2022) and various guilds of raptors (Alivizatos and Goutner 2021; Jaksić and Braker 1983; Piana and Marsden 2012; Silverthorne et al. 2020). There is varied evidence for prey partitioning between raptors. Temporal prey partitioning has been observed in avian scavenger assemblages in Kenya and Spain (Handler et al. 2021; Moreno‐Opo et al. 2016), whereas weak partitioning and a high degree of dietary overlap have been observed between similar species of eagles (Väli et al. 2022) and goshawks (Burton and Olsen 1997). This high degree of dietary overlap can indicate competition; however, a global investigation into five separate raptor assemblages found little evidence of community structure based on competition and that food may not be a limiting resource (Jaksić and Braker 1983). A recent study of three sympatric falcon species reinforced that food may not be a limiting resource, finding minimal differences in habitat use and prey species, with greater dietary overlap than expected by chance (Berlusconi et al. 2025). Some raptors do specialise in particular animal classes, such as Peregrine Falcon ( *Falco peregrinus* ) preying on birds (Bradley and Oliphant 1991; Jenkins and Avery 1999; Razafimanjato et al. 2007) and Osprey ( *Pandion haliaetus* ) on fish (Cartron and Molles 2002; Glass and Watts 2009), whereas some raptors specialise in habitat type, for example, the Harpy Eagle ( *Harpia harpyja* ) hunting arboreal prey with a particular preference for sloths (Aguiar‐Silva et al. 2014). Such specialisms and the evolutionary adaptations to enable them, arise from competitive advantage. They are driven by prey availability and/or competition within a niche, indicating that food is a limiting resource for some raptors. Thus, competition between raptors, and behaviour to reduce it such as resource partitioning, will vary depending on prey availability and the evolutionary potential of the raptor to specialise, enabling them to survive.

Establishing competition and any subsequent prey partitioning between raptor species over large spatial areas can be limited by the intensive resources required to record accurate raptor diets using traditional methods (Bildstein and Bird 2007). In addition, many raptor diets are recorded during the breeding season, creating spatial bias from the focus on nest sites, temporal bias from observing only a portion of the year, and limiting knowledge to breeding individuals (Naude et al. 2019; Panter and Amar 2022). With broad spatial and temporal reach, citizen science is being used in ecology as a year‐round method of monitoring large spatial areas when traditional methods are impractical (Dickinson et al. 2010). Photographs and videos from web‐based sources such as Google Images and citizen science databases, have been used to produce diets for a variety of raptors including caracaras (Panter et al. 2024; Pantoja‐Maggi et al. 2024), Eurasian Sparrowhawks ( *Accipiter nisus* ) (Panter and Amar 2021, 2022), Martial Eagles ( *Polemaetus bellicosus* ) (Naude et al. 2019), Montagu's Harriers ( *Circus pygargus* ) (Kannan et al. 2023) and Tiny Hawks (*Microspizias superciliosus*) (Berryman and Kirwan 2021). Furthermore, diets produced from social media for Eastern Osprey (*P. haliaetus cristatus*) and White‐bellied Sea‐Eagles ( *Haliaeetus leucogaster* ) were shown to be collected an order of magnitude faster than direct visual observations and were successful in removing the spatial and seasonal biases of other methods (Biggs et al. 2025). However, there are potential disadvantages to using web‐based media, including lower species identification rates, spatial bias to areas of greater human activity, reduced spatial accuracy of observations, and a potential bias towards larger or more interesting prey items (Biggs et al. 2025; Naude et al. 2019; Panter et al. 2024). Because of these limitations, this method used in isolation may not be suitable for more advanced dietary studies such as prey partitioning. This study aims to assess the validity of this statement by using citizen science data to investigate potential prey partitioning between two raptors with overlapping distributions in Australia.

**FIGURE 1:**
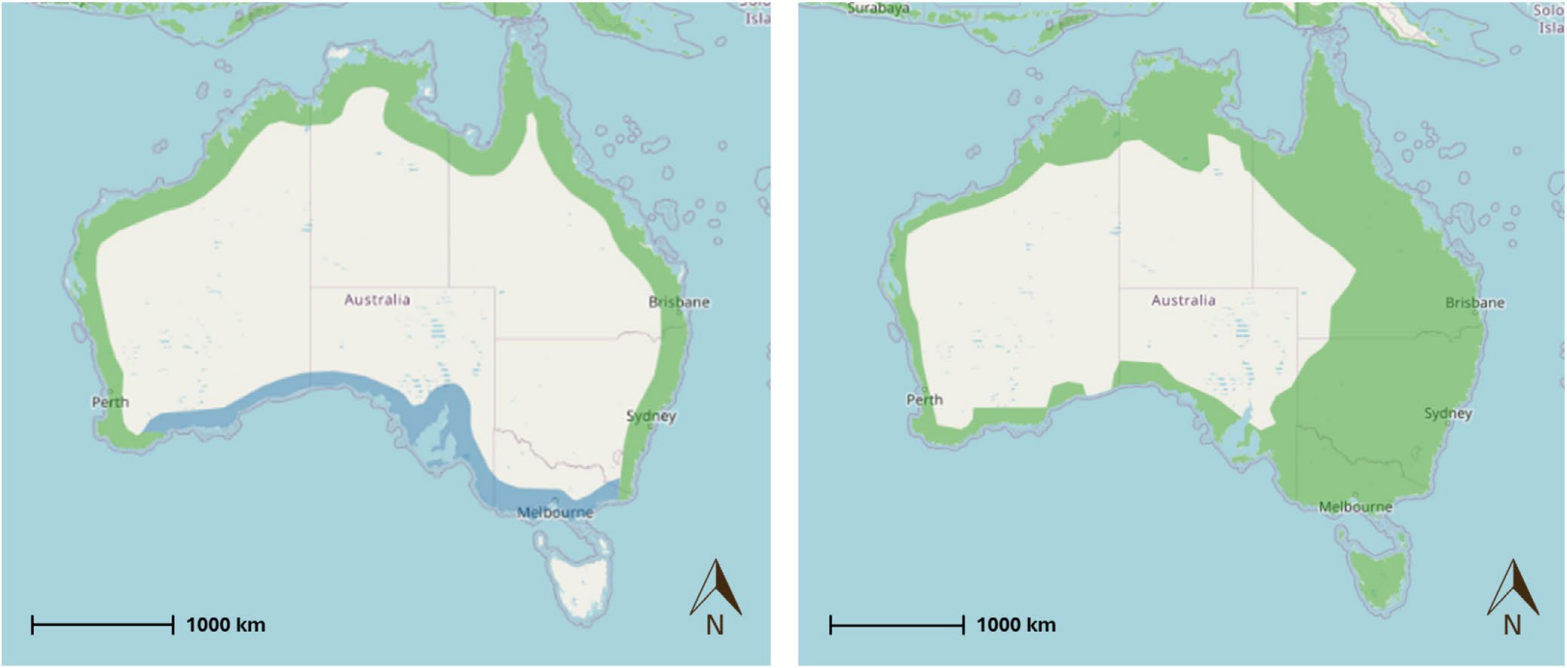
Resident (green) and nonbreeding (blue) range of Eastern Osprey (*Pandion haliaetus cristatus*) (left) and White‐bellied Sea‐Eagle ( *Haliaeetus leucogaster* ) (right) in Australia (Birdlife International 2025).

Whilst determining the value of social media as a method for recording the diets of Eastern Osprey and White‐bellied Sea Eagle (WBSE) in Australia (Biggs et al. 2025), the authors anecdotally noticed potential partitioning in the fish component of the diets. Dietary overlap between pairs of Australian raptors has been investigated between WBSE and Whistling Kite ( *Haliastur sphenurus* ) (Olsen et al. 2013), WBSE and Wedge‐tailed Eagles ( *Aquila audax* ) (Olsen, Fuentes, and Rose 2006), Wedge‐tailed Eagles and Little Eagles ( *Hieraaetus morphnoides* ) (Olsen et al. 2010) and Brown Goshawk (*Tachyspiza fasciata*) and Grey Goshawk ( *T. novaehollandiae* ) (Burton and Olsen 1997). There have also been investigations into the diet of raptor assemblages in the Northern and Australian Capital Territories (Aumann 2001; Aumann et al. 2016; Corbett et al. 2014; Fuentes et al. 2024; Olsen, Fuentes, Rose, and Trost 2006). However, partitioning between Eastern Osprey and WBSE has not been formally evaluated. Nonetheless, a previous study in Queensland also suggested that Eastern Osprey and WBSE may partition fish prey (G. C. Smith 1985). In addition, Eastern Osprey and WBSE have spatial distributions that overlap in Australia (Figure 1), are both considered large raptors with wingspans greater than 1.5 m (Debus 2019), and competition in the form of territorial aggression and kleptoparasitism, that is, stealing of prey, has been observed between them (Dennis and Baxter 2006). As such, there is direct competition between the species with the potential for prey partitioning.

Both Eastern Osprey and WBSE populations in Australia are primarily located in coastal beach, reef and estuarine environments, with some individuals found inland near permanent water bodies such as dams, lakes and rivers (Debus 2019). While Eastern Osprey are known to be fish specialists, WBSE are more opportunistic, supplementing their diet with locally abundant resources such as waterbirds (Olsen et al. 2013) and turtles (Corbett and Hertog 2011). In addition, a coastal study including both species observed WBSE feeding on carrion, while Eastern Osprey did not (Thomson et al. 2016). Osteichthyes (bony fish) is the primary prey class for both species, accounting for 99% of Eastern Osprey diet and 71% of WBSE across Australia (Biggs et al. 2025). Despite this common preference, the species use significantly different methods for hunting fish. Eastern Osprey typically dive feet‐first from around 30 m into the water to capture deeper slow moving fish, whilst WBSE tend to glide and pluck faster‐moving fish from nearer the surface (Debus 2008, 2017, 2019; G. C. Smith 1985). Fish species selection is likely based upon catchability using these techniques, combined with prey abundance and size. WBSE are the larger of the two species weighing between 2.1 and 3.9 kg, versus between 0.8 and 1.9 kg for Eastern Osprey (Debus 2019; Olsen, Fuentes, and Rose 2006). This difference is reflected in the weight of fish caught, with Eastern Osprey prey ranging up to 0.9 kg (Clancy 2005) and WBSE up to 2.5 kg (Debus 2008; Olsen et al. 2013; Olsen, Fuentes, and Rose 2006) with the average fish weighing less than half these values for both raptors. These differences in prey weight and hunting styles further emphasise the potential for prey partitioning between Eastern Osprey and WBSE.

In this study, we used raptor diets and prey abundance data sourced from citizen science resources to investigate prey partitioning of fish between Eastern Osprey and WBSEs in Australia. Dietary breadth, niche overlap, prey diversity and interspecies competition indices were calculated to provide evidence towards the study outcomes.

## 2 Material and Methods

### 2.1 Data Collection, Diet

Data for the Osteichthyes component of Eastern Osprey and WBSE diet was taken from the study by Biggs et al. (2025). That study searched Facebook birding groups and Instagram for posts dated between the 1st January 2019 and 31st December 2023, recording photographs and videos of these raptors in possession of prey items. Across Australia, 1484 Eastern Osprey and 501 WBSE prey items were recorded, but only in New South Wales (NSW) and Queensland (QLD) was the Osteichthyes diet component large enough for comparison (*N* > 30 for both raptor species). The study by Biggs et al. (2025) imputed a large part of the WBSE diet to reduce a bias caused by Osteichthyes being more likely to remain unidentified than other animal classes. However, this imputation was done using animal class and state to predict the missing values. As this study uses just a single animal class and examines individual states, it was not appropriate to use the imputed data, and the smaller original data set was used (Table A1). A total of 33 Osteichthyes prey families was recorded across the two raptor diets.

### 2.2 Data Collection, Prey Abundance

State government recreational fishing catch data were used to calculate the relative abundance of Osteichthyes families in NSW (Murphy et al. 2023) and QLD (Teixeira et al. 2021). These citizen science data were extrapolated from approximately 2000 angler diaries covering a 12‐month period in each state, with participants contacted at random using sampling of state phone numbers. Thus, catch records were distributed throughout each state, but biased by human population density, mirroring the bias of the raptor diet data. NSW data were recorded during the 2021 to 2022 financial year, and QLD data were recorded during the 2019 to 2020 financial year, both falling within the same observation period of the raptor diets. Only the abundance of members of the Osteichthyes families recorded in the raptor diets was included. This approach had the effect of excluding Chondrichthyes (sharks and rays) and fish that may be caught by anglers but not targeted by these raptors, allowing equivalent comparisons.

### 2.3 Dietary Breadth

Dietary breadth for the diets of Eastern Osprey and WBSE in NSW and QLD was calculated using Smith's Niche Measure (E. P. Smith 1982). This metric incorporates the relative abundance of prey and is a standardised measure with values ranging from zero (minimal breadth) to one (maximal breadth). In addition, the number of frequently used resources by each raptor was calculated using a minimum cut‐off value equal to the reciprocal of the total number of resources (1/n) recorded for each Australian state. Furthermore, the Shannon and inverse Simpson diversity indices were calculated for each diet (Shannon 1948; Simpson 1949).

### 2.4 Niche Overlap Assessment

Niche overlap was calculated using percentage overlap (Renkonen 1938, as cited in Krebs 2014) and Morisita's Index (Morisita 1959). Niche overlap measures are susceptible to sample bias when sample sizes are small, uneven between species, or when the quantity of different resources is large (Krebs 2014; Ricklefs and Lau 1980; Smith and Zaret 1982). Unfortunately, this study is impacted by all three of these biases. In a comparison of different niche overlap indices, Morisita's Index (Equation 1) was recommended as the least biased, with near zero bias for small samples and large numbers of different prey resources (Smith and Zaret 1982), and has been used in this study. In contrast, percentage overlap was heavily biased but has also been included as it is a simple, easily understood measure that can provide additional context.

### 2.5 Interspecies Competition Evaluation

Many niche overlap studies are completed with the goal of informing competition; however, a high degree of niche overlap does not necessarily correlate with strong competition (Abrams 1980). Furthermore, it is generally accepted that niche overlap indices cannot be used as competition coefficients (Krebs 2014). Accurate competition calculations require knowledge on the carrying capacity, intrinsic rate of increase and energy density of resources, as well as the maintenance and replacement costs of consumers (MacArthur 1968). Our study was limited by the absence of knowledge in these areas, with only data on the niche overlap and relative abundance of resources available. However, the relative abundance of utilised resources (Table A1) does help inform competition, particularly interspecies competition. A simplistic niche model can be formed if it is assumed that all resources have similar energy density, intrinsic growth and carrying capacity per unit, are distributed equally, and that Eastern Osprey and WBSE have similar population sizes, distributions and energy requirements. Such a model, although limited by these assumptions, can estimate the potential for inter‐species competition within a niche based on the relative abundance of resources. Hurlbert's Index could be used in such situations (Hurlbert 1978), but it uses the relative abundance of all resources regardless of whether they are utilised. Instead, we modified the low bias Morisita's Index, dividing by the relative abundance of utilised resources (Equation 1). The modified index works on the established principle that interspecies competition is lower for abundant resources (Abrams 1980; Hurlbert 1978). Both Morisita's index, and the interspecies competition index derived from it, are not normalised indexes and can produce values greater than one. This can occur in cases where sample sizes are very uneven and shared dominant prey items inflate the score. Index values range from zero (minimal competition) to an undefined maximum that is typically below 2.

(1) CI=2∑inpijpikai∑inpijnij−1Nj−1ai+∑inpiknik−1Nk−1ai

where *a*_*i*_, Relative abundance of resource *i* to all utilised resources; *C*_*I*_, Interspecies competition index; *n*, Total number of resource states (*i* = 1, 2, 3, …… *n*); *p*_*ij*_, Proportion resource *i* is of the total resources used by species *j*; *p*_*ij*_, Proportion resource *i* is of the total resources used by species *k* (Equation 1). Interspecies competition index, derived by modifying Morisita's niche overlap index to include the relative abundance of resources.

### 2.6 Statistical Analyses

The observed Morisita's and inter‐species competition index values were compared to diets formulated by chance. Diets for NSW and QLD with the same sample size per raptor as the observed diets, were formulated through random prey assignment. Morisita's and intercompetition values were calculated, and the process was repeated 10,000 times to form null distributions. *p* values were calculated to indicate how many times the null values were greater than or equal to the observed values. The observed relative abundance values were used when calculating interspecies competition, and prey items with zero abundance in the angler surveys were excluded.

## 3 Results

### 3.1 Dietary Breadth

A broad and even diet was recorded for both Eastern Osprey and WBSE in both NSW and QLD (Table 1). A total of 18 Osteichthyes families was observed between the diets of the two raptors in NSW, and 28 in QLD. Using the reciprocals of these values gave minimum diet abundance cut‐offs for the quantity of frequently used species as 5.6% and 3.6%, respectively. Prey abundance data were available for all Osteichthyes families recorded in the diets, except for Cichlidae (tilapia) in QLD and Scatophagidae (scats) in both NSW and QLD.

**TABLE 1:** Dietary breadth metrics for Eastern Osprey (*Pandion haliaetus cristatus*) and White‐bellied Sea Eagle (WBSE, *Haliaeetus leucogaster* ) in New South Wales and Queensland, Australia.

	New South Wales		Queensland	
	WBSE	Eastern Osprey	WBSE	Eastern Osprey
Number of Osteichthyes prey	53	156	35	310
Number of prey families	14	9	16	27
Number of frequently used resources	6	5	7	5
Evenness	0.89	0.72	0.91	0.69
Shannon diversity	2.35	1.58	2.52	2.27
Inverse Simpson diversity	8.59	3.79	9.96	5.90
Smith's niche breadth	0.61	0.69	0.72	0.75

Pearson's correlation was calculated at a 95% confidence level between the dietary proportion of Osteichthyes families for each raptor and their relative abundance. In NSW, diet proportions for both raptors had a nonsignificant weak positive correlation to relative abundance (Eastern Osprey: *r*(16) = 0.26, *p* = 0.29; WBSE: *r*(16) = 0.10, *p* = 0.69). In QLD, diet proportions had a moderate positive correlation to relative abundance with borderline levels of significance (Eastern Osprey: *r*(16) = 0.40, *p* = 0.04; WBSE: *r*(16) = 0.37, *p* = 0.05).

### 3.2 Niche Overlap and Inter‐Species Competition

**FIGURE 2:**
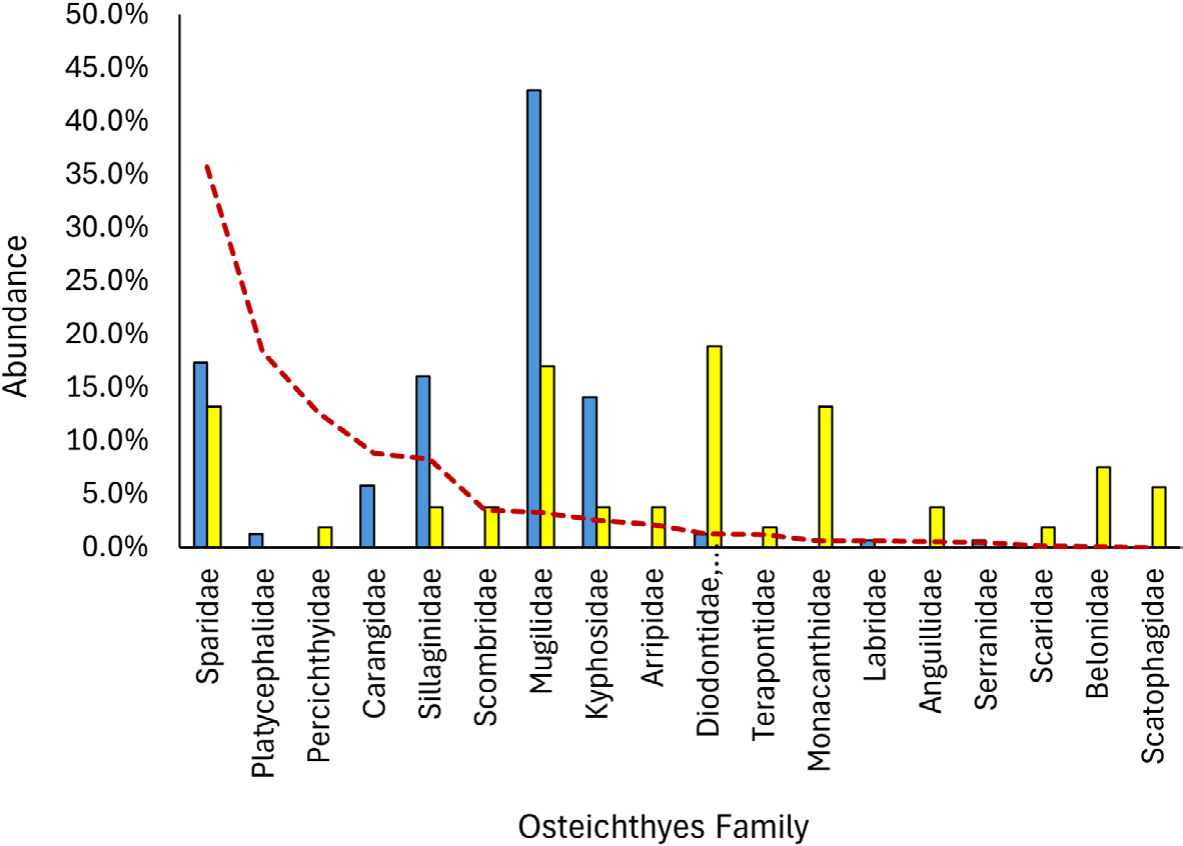
Abundance of Osteichthyes families in Eastern Osprey (*Pandion haliaetus cristatus*) (blue) and White‐bellied Sea‐Eagle ( *Haliaeetus leucogaster* ) (yellow) diets and their relative abundance (dotted red line), recorded across the state of New South Wales, Australia.

**FIGURE 3:**
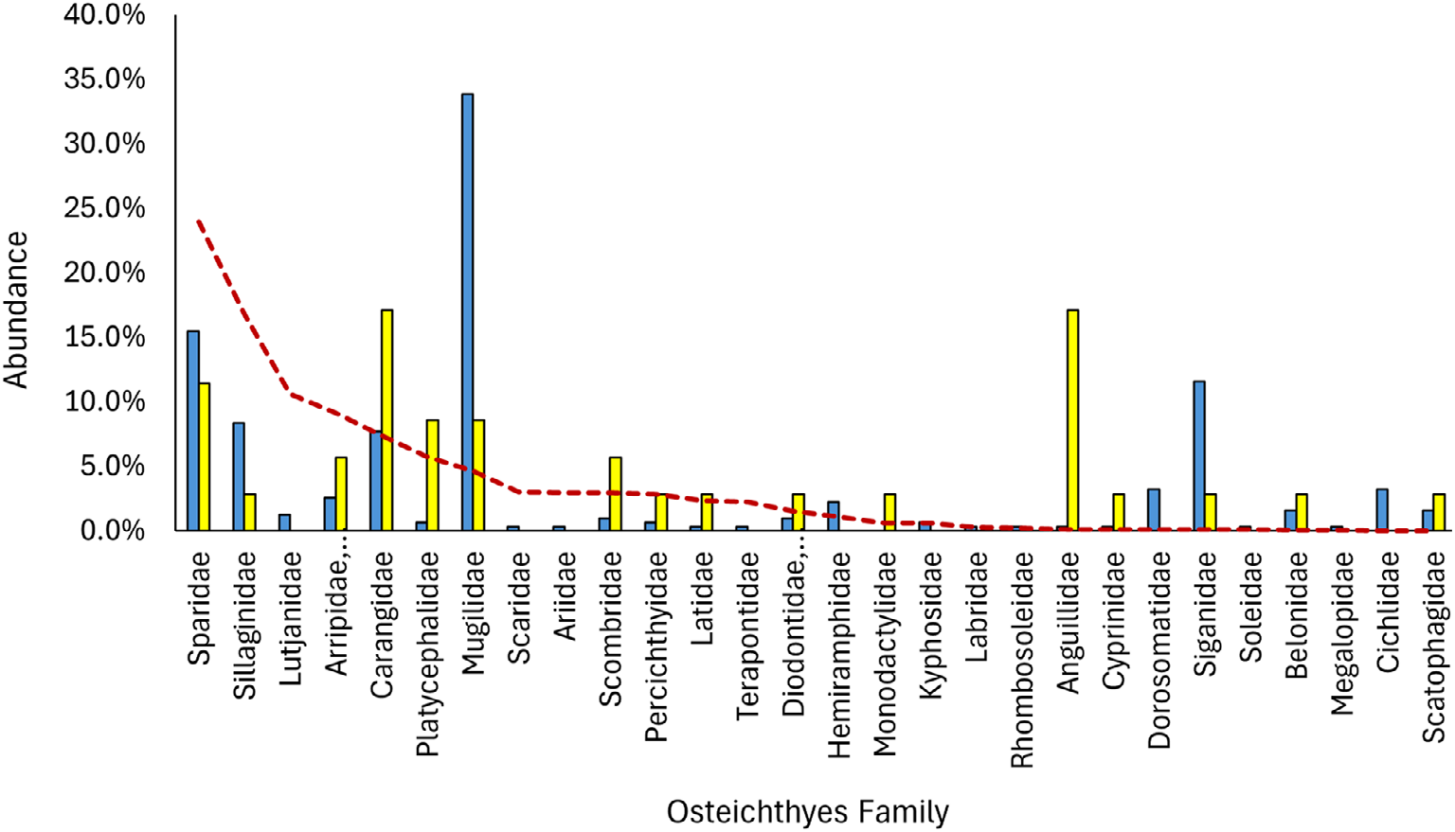
Abundance of Osteichthyes families in Eastern Osprey (*Pandion haliaetus cristatus*) (blue) and White‐bellied Sea‐Eagle ( *Haliaeetus leucogaster* ) (yellow) diets, and their relative abundance (dotted red line), recorded across the state of Queensland, Australia.

**FIGURE 4:**
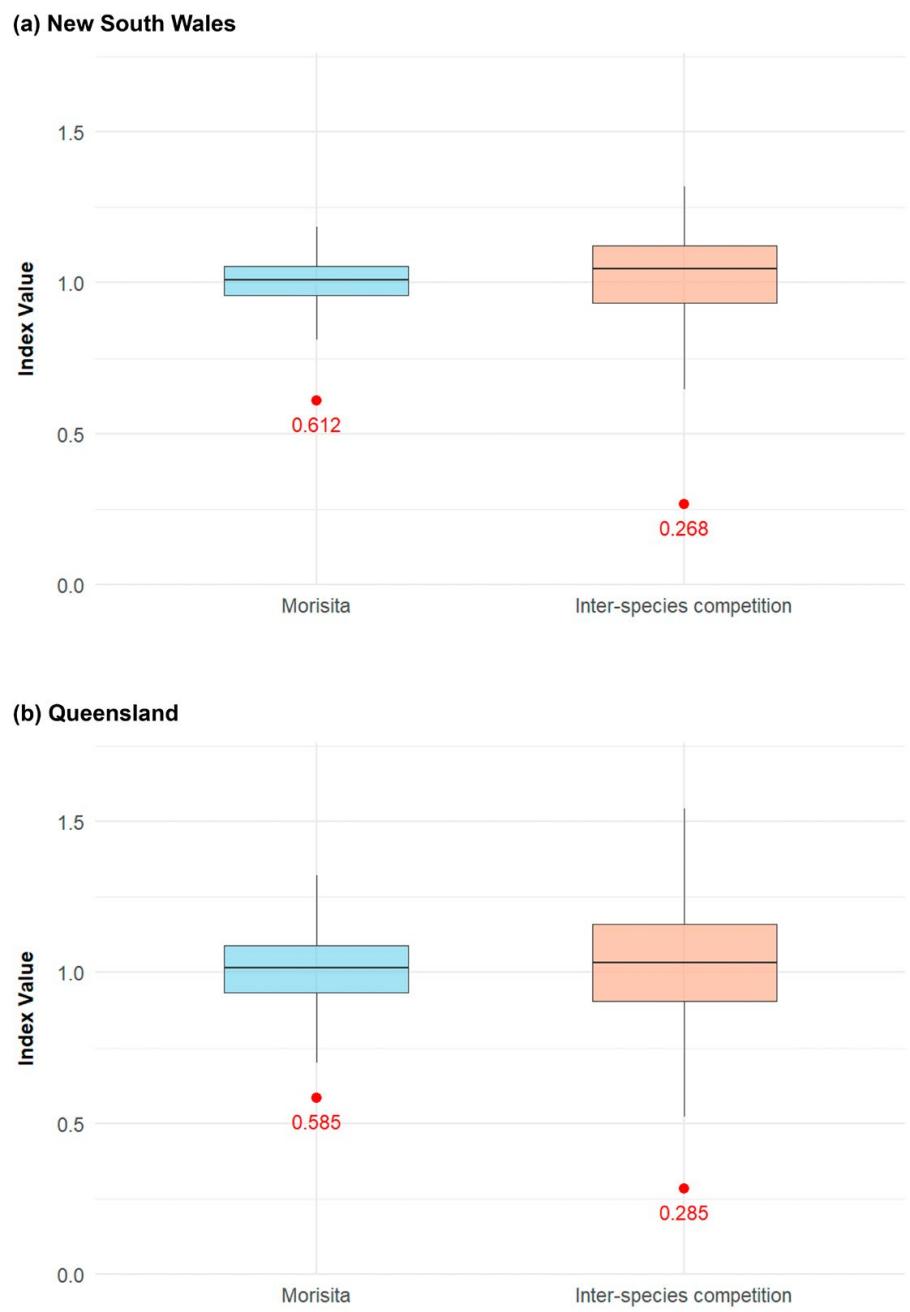
Null distribution of Morisita's niche overlap index and interspecies competition index values between Eastern Osprey (*Pandion haliaetus cristatus*) and White‐bellied Sea‐Eagle ( *Haliaeetus leucogaster* ) diets generated by chance in (a) New South Wales and (b) Queensland, Australia. Observed values from the study are provided in red.

The percentage overlap between the Eastern Osprey and WBSE diets was 39.0% in NSW and 43.5% in QLD. In total, 5/18 Osteichthyes families overlapped in NSW (Figure 2) and 15/28 in QLD (Figure 3). The greatest overlaps were present in the Sparidae (bream, snapper), Mugilidae (mullet) and Carangidae (trevally, scad, etc.) prey families. Morisita's niche overlap index values of 0.61 and 0.59 were recorded in NSW and QLD respectively, indicating a moderately overlapping diet. Interspecies competition index values of 0.27 and 0.29 were recorded in NSW and QLD respectively, suggesting a low level of competition between the two species. In both NSW and QLD, the Morisita's niche overlap index and interspecies competition index values were below the range of values from diets generated by chance (Figure 4), indicative of a partitioned diet with reduced competition.

## 4 Discussion

### 4.1 Dietary Breadth and Diversity

Dietary diversity was slightly higher for WBSE than Eastern Osprey, because of a lower evenness in the Eastern Osprey diet caused by the dominant mullet component. Variances in the total number of prey species observed in each raptor diet did not translate to differences in the total number of frequently used resources. In addition, the Smith index for niche breadth was similar between the two raptor species. Overall, there was little difference in dietary breadth and diversity between the two species and both diets could be described as moderately broad and diverse.

However, there were slight differences between states, with the QLD diets for both raptors being broader and more diverse than in NSW. This difference was not surprising as average water temperatures in QLD are higher than those in NSW. Most fishes are ectothermic, and higher water temperatures aid metabolism and growth, leading to greater species richness up until the point at which water temperature limits which species can survive (Wootton 1992). Higher water temperatures also lead to a diversification in habitats; this pattern is evident in QLD with the presence of the Great Barrier Reef, an area of high fish diversity with over 1500 species (UNESCO 2025). However, sea warming from climate change has and is likely to further change fish assemblages and distributions throughout Australia, with fish moving to stay within their preferred temperature range (Last et al. 2011).

### 4.2 Dietary Proportion and Prey Abundance

There were nonsignificant weak positive correlations between prey abundance and diet proportion for both raptors in NSW. In contrast, there were marginally significant moderate positive correlations in QLD for both raptors. This difference suggests that QLD raptors may be more responsive to prey abundance, matching their dietary proportions to the abundance of each prey species in their environment. However, there were differences in which Osteichthyes families were most abundant in each state, and it may be that the highly abundant NSW families are harder for the two raptors to catch. Furthermore, it is possible that the behaviour of Osteichthyes families and the species within them varies between each state, for example, inhabiting water depths out of reach of both raptors.

### 4.3 Dietary Overlap and Prey Abundance

Morisita's niche overlap values for both NSW and QLD indicated a moderate overlap in diet. This overlap appeared to be driven by a high degree of overlap in some highly abundant Osteichthyes families, suggesting a relationship between prey abundance and dietary overlap between Eastern Osprey and WBSE. In NSW, the diet showed greatest overlap in the Sparidae and Mugilidae families. Substantial overlap of these two families was also observed in QLD, as was Carangidae. Most Sparidae observations were of bream species, particularly Yellowfin Bream ( *Acanthopagrus australis* ) and of snapper species. This family was the most abundant in both states, accounting for 36% of Osteichthyes in NSW and 24% in QLD. Due to this high abundance, it is likely that Eastern Osprey and WBSE are not in competition for this family, explaining the overlap.

Mugilidae, known commonly as mullets, comprise several species, but predominantly Sea Mullet ( *Mugil cephalus* ). Mullets were not caught in high abundance in the recreational anglers' surveys, because their diet comprises microcrustaceans which limits line fishing (Prosser 2016; Smith and Deguara 2002). However, commercial catch estimates of 2938 t in NSW and 1164 t in QLD for 2020 using nets (Lovett et al. 2022), accounted for 18% and 4% of commercially caught seafood in each state respectively (Tuynman and Dylewski 2022), suggesting a high abundance. However, even factoring in this higher relative abundance, there was a clear preference for mullet in the Eastern Osprey diet, accounting for 43% in NSW and 34% in QLD. Reasons for this preference include that Sea Mullets often weigh up to 2 kg, have a streamlined body shape which is easy to carry, swim in open water in the middle to upper portion of the water column and form vast shoals in Eastern Australia during their spawning season (DWER 2025; Prokop 2006). Sea Mullets are ideal prey for both raptor species, but particularly Eastern Osprey which can dive into the water to reach a greater portion of the water column. Due to the high abundance of mullets, again it is unlikely that Eastern Osprey and WBSE compete for them, resulting in dietary overlap.

The overlap of Carangidae in QLD appears driven by darts, also known as pompano (*Trachinotus* sp.), accounting for 57% and 46% of Carangids caught by WBSE and Eastern Osprey respectively. Carangidae were the fifth most frequently caught Osteichthyes by QLD anglers, with over 700,000 caught and a relative abundance of 9.2%. Furthermore, a third of these were attributed to dart species. Australian dart species typically weigh up to 1 kg, are often found in reefs near the surf zone of sandy beaches, and can be found swimming in pairs or small groups in surface water (Kuiter and Tonozuka 2004; Prokop 2006). This high availability in both abundance and ease of access makes darts a prime target for both Eastern Osprey and WBSE.

However, high prey abundance did not always correspond to high consumption in these raptors' diets. Some highly abundant Osteichthyes families may be difficult to catch for either or both raptor species, particularly benthic feeders such as Lutjanidae (snappers) that feed on crustaceans on the sea floor. This was observed in our data, with a high abundance but low dietary contribution. In addition, the physiology of some families such as Platycephalidae (flatheads) may be unsuitable for inclusion in the raptor diets. Flatheads were highly abundant in the angler surveys, but only WBSE in QLD caught this prey at a similar rate to abundance. Very few flatheads were caught by Eastern Osprey. The most frequently caught flathead by anglers was the Dusky Flathead ( *Platycephalus fuscus* ), the largest flathead species in Australia weighing up to 15 kg (DPI (Department of Primary Industries) 2025; Prokop 2006), with an average of 0.9 kg (Yang et al. 2022). Thus, only the juvenile portion of the population would be suitable for Osprey, potentially explaining the low dietary share. It is less clear why WBSE did not catch flatheads in NSW.

### 4.4 Inter‐Species Competition, Diet Partitioning and Specialism

The low interspecies competition index values reflected the aforementioned high abundance of the overlapping Osteichthyes families. This result mirrors what has previously been suggested in that competition can be lower with higher niche overlap if the resource is in high abundance (Abrams 1980; Hurlbert 1978). In addition, a large quantity of Osteichthyes families was caught exclusively by either Eastern Osprey or WBSE, contributing zero overlap or competition (13/18 in NSW and 13/28 in QLD). Furthermore, some families were utilised more frequently by one raptor species than the other. Examples in NSW included Kyphosidae (Luderick) and Sillaginidae (whiting), accounting for 30% combined of the Eastern Osprey diet compared to just 8% of the WBSE diet. This difference is likely because both of these families forage on the ocean bed (DPI (Department of Primary Industries) 2025), thus the diving capability of Eastern Osprey is required to reach them. Conversely, Diodontidae (porcupinefish) and Tetraodontidae (toadfish) accounted for a combined 1% of Eastern Osprey diet compared to 19% of WBSE diet. This example highlights a WBSE specialism as porcupinefish and toadfish have significant quantities of neurotoxins in their skin (Halstead 1965), and WBSE have learnt to selectively dissect these prey or have developed an immunity to the toxins (Biggs et al. 2025; G. C. Smith 1985).

Similar disparities were present in QLD with examples including Anguillidae (freshwater eels) (0.3% Eastern Osprey vs. 17% WBSE) and Siganidae (Happy Moments) (12% Eastern Osprey vs. 4% WBSE). Freshwater eels often inhabit slow‐moving or still water (McDowall and Beumer 1980; Prokop 2006), making them available to either raptor species. However, freshwater eels are large, with the smaller of the two common species, Southern Shortfin Eel ( *Anguilla australis* ), growing to 0.9 m (McGrouther 2020), making it potentially unsuitable for Eastern Osprey. In contrast, Happy Moments are herbivorous, feeding on the sea floor on a diet of algae and sea grass (Bray 2020), making them hard for WBSE to hunt but within the hunting range of Eastern Osprey. It is interesting to note that no Happy Moments were caught by either raptor in NSW, despite their range extending to the southern border of the state (McGrouther 2021).

These examples of less abundant Osteichthyes families suggest a level of prey partitioning between Eastern Osprey and WBSE based on the availability of prey species and raptor hunting method. This pattern of lower prey abundance and raptor diet partitioning is reinforced when examining the percentage overlap of the Eastern Osprey and WBSE diets. The three highly abundant and overlapping families (Carangidae, Mugilidae, Sparidae) accounted for 78% of the dietary overlap in NSW and 64% in QLD. There was minimal overlap in the remaining families.

**FIGURE 5:**
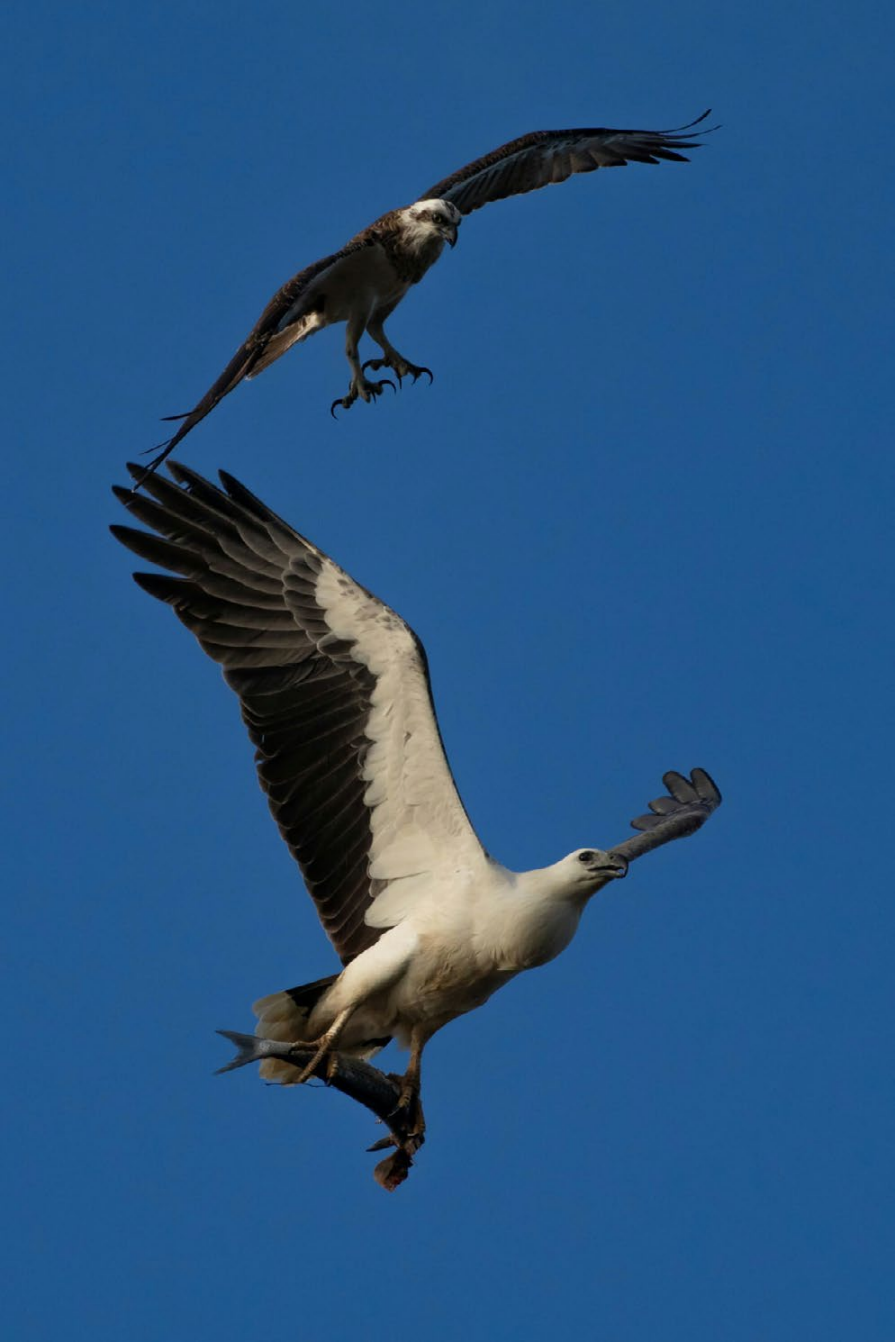
Example of kleptoparasitic behaviour between Eastern Osprey (*Pandion haliaetus cristatus*) and White‐bellied Sea‐Eagle ( *Haliaeetus leucogaster* ) observed at Peel Inlet, Mandurah, Western Australia (Fenton 2020).

Despite the indication of partial partitioning and minimal competition between the raptor species, there were five instances of kleptoparasitism observed between them (Figure 5). Combined with other recorded observations of this behaviour (Cupper and Cupper 1981; Dennis and Baxter 2006), it is apparent that the species are in direct competition.

### 4.5 Do the Citizen Science Resources Provide Everything Required to Demonstrate Partitioning?

The observed Morisita's niche overlap index values were shown to be below the distribution that would be expected if the two raptors were selecting their diet randomly. This is evidence that the two raptor species partition their diets. However, further data are required to conclusively understand competition between the species through the completion of established competition formulae (Abrams 1980; MacArthur 1968) and confirm any hypotheses for diet partitioning. However, when considering the efficiency improvements of using citizen science, researchers would have more available time to record these data and make thorough competition assessments, providing the necessary knowledge to conclusively evaluate prey partitioning.

There were also concerns about the taxonomic detail provided by using social media for prey identification, with most prey only identifiable to the family level. When researching fish biology to explain overlap and differences in the raptor diets, we found most species within the Australian Osteichthyes prey families behaved in similar ways, utilising similar areas of the niche to consume food. We also had some species level data for both the relative abundance of available prey, and also which species were more frequently caught by the raptors. As such, it was possible to provide context as to why some families were consumed by one raptor species and not the other by considering the biology of the most frequently consumed species. However, species level taxonomic detail may be required in other studies where predators hunt other animal classes that vary more in behaviour within families.

### 4.6 Limitations

There are biases in the diet data we used, which have been detailed thoroughly by Biggs et al. (2025). One of the most important biases for this study was the potential overestimation of easily identifiable or ‘interesting’ prey. It is likely that the unidentified Osteichthyes excluded from this study would have a higher proportion of fish with fewer identifiable features, such as mullet. This factor could potentially skew the preference of Eastern Osprey further towards mullet, increase the dietary overlap between Eastern Osprey and WBSE, and reduce the diversity of the raptor diets. However, there could equally be other hard‐to‐identify Osteichthyes families which have not been included in this study. This absence would have the reverse effect, decreasing dietary overlap and increasing dietary diversity.

Another limitation was the lack of refined spatial information for the raptor diet observations. This lack meant that we compared diet across whole Australian states, and it is possible that the observations for each raptor came from completely different areas. However, this is very unlikely to be true as kleptoparasitism between the two species was observed in the data, and previous studies and citizen science databases have shown them inhabiting the same areas (Fink et al. 2025; G. C. Smith 1985). We also lacked spatial data for the prey relative abundance data, which were likely biased towards NSW and QLD cities where the anglers are more likely to live. Although WBSE and Eastern Osprey inhabit these same areas, and a similar bias is likely in the raptor diet observation data, it is impossible to determine the similarity of the dataset location biases.

There was also potential bias caused by the inequity in sample size between Eastern Osprey and WBSE diets. Because the WBSE sample was substantially smaller, it is possible that not all prey species are present. This imbalance indicates that the diversity and breadth of the WBSE diet are likely to be underestimated in this study.

Furthermore, the angler data may have significant bias. Anglers choose to target specific fish based on a variety of variables including taste, size, ease of access, bait availability, spikes in abundance, seasons and traditions. As such, although this data provides a reasonable proxy for fish abundance, it is not necessarily accurate. However, it is currently the most efficient method for estimating fish assemblages over such large spatial areas.

## 5 Conclusions and Recommendations for Future Research

Dietary overlap between Eastern Osprey and WBSE was moderate, while interspecies competition was low due to a high abundance of shared prey, and limited overlap in other prey. Despite this situation, direct competition was observed between the species, and a strong, evidence‐led hypothesis was formed that prey partitioning is determined by resource availability. Highly abundant prey was shared, whilst less abundant prey was partitioned according to hunting style, with WBSE swooping to collect fast‐moving fish from the water surface, and Eastern Osprey diving to access deeper slower‐moving fish. The citizen science data used provided adequate detail to understand this relationship. However, additional data are required to comprehensively calculate interspecies competition and conclusively prove the relationship. Future research collating information on the carrying capacity, intrinsic rate of increase and energy density of Australian Osteichthyes is required, as is the measurement of the maintenance and replacement costs of Eastern Osprey and WBSE. In addition, the distribution and abundance of both raptors in NSW and QLD would need to be determined. Furthermore, more accurate spatial information of prey observations would enable more complex dietary dynamics questions to be assessed, such as if WBSE alter the proportion of other animal classes in their diet in response to increased competition for Osteichthyes. Overall, citizen science has been shown to increase the efficiency of recording diets, but future research should use some of the time saved to add conservation value through understanding the ecological niche of species and competition between them.

## Author Contributions

**Leo Biggs:** conceptualization (lead), data curation (lead), formal analysis (lead), investigation (lead), methodology (lead), project administration (lead), software (lead), supervision (lead), writing – original draft (lead), writing – review and editing (equal). **Greg S. Baxter:** conceptualization (equal), formal analysis (supporting), methodology (equal), project administration (equal), supervision (equal), writing – original draft (equal), writing – review and editing (equal). **Stephen J. S. Debus:** conceptualization (equal), formal analysis (supporting), methodology (equal), project administration (equal), supervision (equal), writing – original draft (equal), writing – review and editing (equal). **Neal Finch:** conceptualization (equal), formal analysis (supporting), methodology (equal), project administration (equal), supervision (equal), writing – original draft (equal), writing – review and editing (equal). **Anysha Riggs:** data curation (equal), writing – review and editing (supporting). **Hayden Houweling:** data curation (equal), writing – review and editing (supporting). **Melissa Appleby:** data curation (equal), writing – review and editing (supporting). **Peter J. Murray:** conceptualization (equal), data curation (equal), formal analysis (supporting), investigation (supporting), methodology (equal), project administration (lead), supervision (lead), writing – original draft (equal), writing – review and editing (equal).

## Conflicts of Interest

The authors declare no conflicts of interest.

## Data Availability

All the required data are uploaded as Appendix 1.

## Appendix 1

**TABLE A1:** Relative abundance of utilised Osteichthyes families in Eastern Osprey (*Pandion haliaetus cristatus*) diets, White‐bellied Sea‐Eagle (WBSE, *Haliaeetus leucogaster* ) diets and angler surveys in New South Wales and Queensland, Australia.

		New South Wales			Queensland		
Prey family	Common name(s)	Eastern Osprey	WBSE	Angler survey	Eastern Osprey	WBSE	Angler survey
		*n* = 156	*n* = 53	*n* = 4,067,152	*n* = 310	*n* = 35	*n* = 7,660,000
Anguillidae	Freshwater eel		3.8%	0.5%	0.3%	17.1%	0.1%
Ariidae	Catfish				0.3%		3.0%
Arripidae, Chirocentridae, Clupeidae	Australian Salmon, herring		3.8%	2.2%	2.6%	5.7%	9.2%
Belonidae	Longtom		7.5%	0.1%	1.6%	2.9%	0.1%
Carangidae	Dart, kingfish, queenfish, scad, trevally	5.8%		8.8%	7.7%	17.1%	7.4%
Cichlidae	Tilapia				3.2%		
Cyprinidae	Carp				0.3%	2.9%	0.1%
Diodontidae, Tetraodontidae	Porcupinefish, toadfish	1.3%	18.9%	1.3%	1.0%	2.9%	1.6%
Dorosomatidae	Bony Bream				3.2%		0.1%
Hemiramphidae	Garfish				2.3%		1.1%
Kyphosidae	Luderick	14.1%	3.8%	2.5%	0.6%		0.6%
Labridae	Wrasse	0.6%		0.6%	0.3%		0.3%
Latidae	Barramundi				0.3%	2.9%	2.3%
Lutjanidae	Jack, snapper				1.3%		10.6%
Megalopidae	Tarpon				0.3%		0.1%
Monacanthidae	Leatherjacket		13.2%	0.6%			
Monodactylidae	Butter Bream					2.9%	0.6%
Mugilidae	Mullet	42.9%	17.0%	3.3%	33.9%	8.6%	4.6%
Percichthyidae	Temperate perch		1.9%	12.6%	0.6%	2.9%	2.9%
Platycephalidae	Flathead	1.3%		18.4%	0.6%	8.6%	5.7%
Rhombosoleidae	Flounder				0.3%		0.2%
Scaridae	Parrotfish		1.9%	0.1%	0.3%		3.0%
Scatophagidae	Scat		5.7%		1.6%	2.9%	0.0%
Scombridae	Mackerel, bonito		3.8%	3.5%	1.0%	5.7%	3.0%
Serranidae	Grouper	0.6%	0.0%	0.4%			
Siganidae	Happy Moment				11.6%	2.9%	0.1%
Sillaginidae	Whiting	16.0%	3.8%	8.3%	8.4%	2.9%	16.9%
Soleidae	Sole				0.3%		0.1%
Sparidae	Bream, snapper	17.3%	13.2%	35.7%	15.5%	11.4%	24.0%
Terapontidae	Perch, trumpeter		1.9%	1.2%	0.3%		2.3%
